# Long working hours and change in body weight: analysis of individual-participant data from 19 cohort studies

**DOI:** 10.1038/s41366-019-0480-3

**Published:** 2019-11-25

**Authors:** Marianna Virtanen, Markus Jokela, Tea Lallukka, Linda Magnusson Hanson, Jaana Pentti, Solja T. Nyberg, Lars Alfredsson, G. David Batty, Annalisa Casini, Els Clays, Dirk DeBacquer, Jenni Ervasti, Eleonor Fransson, Jaana I. Halonen, Jenny Head, France Kittel, Anders Knutsson, Constanze Leineweber, Maria Nordin, Tuula Oksanen, Olli Pietiläinen, Ossi Rahkonen, Paula Salo, Archana Singh-Manoux, Sari Stenholm, Sakari B. Suominen, Töres Theorell, Jussi Vahtera, Peter Westerholm, Hugo Westerlund, Mika Kivimäki

**Affiliations:** 1 School of Educational Sciences and Psychology, University of Eastern Finland, Joensuu, Finland; 2 Stress Research Institute, Stockholm University, Stockholm, Sweden; 3 Department of Psychology and Logopedics, University of Helsinki, Helsinki, Finland; 4 Finnish Institute of Occupational Health, Helsinki, Finland; 5 Department of Public Health, Clinicum, University of Helsinki, Helsinki, Finland; 6 Department of Public Health, University of Turku and Turku University Hospital, Turku, Finland; 7 Centre for Population Health Research, University of Turku and Turku University Hospital, Turku, Finland; 8 Institute of Environmental Medicine, Karolinska Institutet, Stockholm, Sweden; 9 Centre for Occupational and Environmental Medicine, Stockholm County Council, Stockholm, Sweden; 10 Department of Epidemiology & Public Health, University College London, London, UK; 11 School of Biological & Population Health Sciences, Oregon State University, Corvallis, USA; 12 IPSY, Université catholique de Louvain (UCLouvain), Louvain-la-Neuve & School of Public Health, Université libre de Bruxelles (ULB), Brussels, Belgium; 13 Department of Public Health, Ghent University, Ghent, Belgium; 14 School of Health and Welfare, Jönköping University, Jönköping, Sweden; 15 Department of Health Sciences, Mid Sweden University, Sundsvall, Sweden; 16 Department of Psychology, Umeå University, Umeå, Sweden; 17 Department of Psychology, University of Turku, Turku, Finland; 18 INSERM, U 1018 Villejuif, France; 19 Folkhälsan Research Center, Helsinki, Finland; 20 University of Skövde, Skövde, Sweden; 21 Occupational and Environmental Medicine, Uppsala University, Uppsala, Sweden

**Keywords:** Risk factors, Preventive medicine

## Abstract

**Objective:**

To examine the relation between long working hours and change in body mass index (BMI).

**Methods:**

We performed random effects meta-analyses using individual-participant data from 19 cohort studies from Europe, US and Australia (*n* = 122,078), with a mean of 4.4-year follow-up. Working hours were measured at baseline and categorised as part time (<35 h/week), standard weekly hours (35–40 h, reference), 41–48 h, 49–54 h and ≥55 h/week (long working hours). There were four outcomes at follow-up: (1) overweight/obesity (BMI ≥ 25 kg/m^2^) or (2) overweight (BMI 25–29.9 kg/m^2^) among participants without overweight/obesity at baseline; (3) obesity (BMI ≥ 30 kg/m^2^) among participants with overweight at baseline, and (4) weight loss among participants with obesity at baseline.

**Results:**

Of the 61,143 participants without overweight/obesity at baseline, 20.2% had overweight/obesity at follow-up. Compared with standard weekly working hours, the age-, sex- and socioeconomic status-adjusted relative risk (RR) of overweight/obesity was 0.95 (95% CI 0.90–1.00) for part-time work, 1.07 (1.02–1.12) for 41–48 weekly working hours, 1.09 (1.03–1.16) for 49–54 h and 1.17 (1.08–1.27) for long working hours (*P* for trend <0.0001). The findings were similar after multivariable adjustment and in subgroup analyses. Long working hours were associated with an excess risk of shift from normal weight to overweight rather than from overweight to obesity. Long working hours were not associated with weight loss among participants with obesity.

**Conclusions:**

This analysis of large individual-participant data suggests a small excess risk of overweight among the healthy-weight people who work long hours.

## Introduction

Obesity is a modifiable risk factor for an array of health problems, including cardiovascular disease, type 2 diabetes, certain cancers and dementia that collectively contribute to a substantial proportion of disease burden and death worldwide [1, 2]. Resulting also from changes in living and working environments, the global ‘epidemic’ of obesity currently affects all age groups, all populations, and countries of all income levels [3, 4].

Psychosocial work environment may have a role in weight control although little research has been published beyond perceived stress at work. Current evidence suggests a modest association between work stress and overweight or obesity, with limited support from longitudinal studies [5, 6]. Another work-related aspect, long working hours, may also contribute to weight gain, owing to extended periods of sitting, reduced opportunities for exercise, changes in eating habits leading to positive energy balance, and subsequently to weight gain.

Previous studies suggest associations of long working hours with increased incidence of cardiovascular disease and type 2 diabetes [7–9], for which obesity is also a major risk factor [10, 11]. There are few prospective studies examining the link between long working hours and changes in body weight, and the findings are inconsistent with positive [12–15], and null [16] associations reported. Further, one study found that long working hours may confer protection against gaining weight [17]. In addition to the paucity of evidence, studies in this field are typically insufficiently powered to examine weight gain amongst people who are previously not overweight.

Accordingly, with a large collaborative study of 19 cohorts from Europe, the US and Australia, we examined the association between working hours and subsequent change in body mass index (BMI), including an analysis of long working hours in relation to each stage of weight gain: from non-overweight to overweight or obesity, from overweight to obesity. Long working hours may also be associated with lower probability of weight loss among those with obesity. For the first time to our knowledge, the size of this dataset allows to examine also this issue.

## Materials and methods

### Participants

We used data from 19 cohort studies (Supplementary Table 1) which were either taken from Individual-Participant-Data Meta-analysis in Working Populations (IPD-Work) Consortium [18], or were available from one of two digital repositories of research data (the Inter-University Consortium for Political and Social Research; http://www.icpsr.umich.edu/icpsrweb/ICPSR/) or the UK Data Service (http://ukdataservice.ac.uk/). The studies were chosen according to the availability of working hours and BMI data in a prospective cohort design with the follow-up not exceeding 10 years. All studies have their own approval from their relevant local or national ethics committee, and all participants had given informed consent.

Six cohort studies were from the UK: British Birth Cohort 1970 (BCS1970) [19], British Household Panel Survey [20], National Child Development Study [21], Whitehall II study [22], UK Household Longitudinal Study [23], and English Longitudinal Study of Aging [24]; four were from the US: Americans’ Changing Lives [25], Midlife in the United States [26], Health and Retirement Study [27], and National Longitudinal Survey of Youth [28]; three from Finland: Finnish Public Sector Study [29], Health and Social Support Study [30], and Helsinki Health Study (HHS) [31]; two from Sweden: Swedish Longitudinal Occupational Survey of Health [32] and Work, Lipids and Fibrinogen Norrland [33]; and one each from Belgium: Belgian Job Stress Project (Belstress) [34]; Germany: German Socioeconomic Panel Survey [35]; and Australia: Household, Income, and Labour Dynamics in Australia [36]. In addition, we included the Survey of Health, Ageing and Retirement in Europe [37], which included data from multiple European countries (France, Germany, Finland, Sweden, Denmark, Slovenia, Cyprus, Romania, Bulgaria, Greece, Czech Republic, Slovakia, Hungary, Estonia, Latvia, Lithuania, Poland, The Netherlands, Belgium, Luxembourg, Spain, Portugal, Italy, Switzerland, and Austria).

In total, there were 122,078 participants for prospective analyses who were at work and provided data on working hours, sociodemographic characteristics and repeat measurements of BMI.

### Assessment of working hours

In all cohorts, information on working hours at baseline was obtained from self-report. We used the previously used categorisation of weekly working hours: <35 (part time), 35–40 (reference group), 41–48, 49–54 and ≥55 h (long working hours) [38, 39]. In all these studies, working hours data were based on a continuous response, the only exception being HHS [31] where responses were based on a pre-defined categorises (<30, 30–40, 41–50 and >50 h/week). For the purposes of the present analyses, we treated in that study the category of 30–40 h/week as the reference with <30 h/week corresponding to part time, 41–50 h/week corresponding to the 41–48 h category in meta-analysis and >50 working hours corresponding to the ≥55 h category.

### Assessment of body mass index (BMI)

At baseline and follow-up, BMI (expressed as kg/m^2^) was based on self-reported height and weight in all cohort studies except Whitehall II and Belstress, in which height and weight were measured directly during a standardised clinical examination. The following categories at baseline and at follow-up were calculated: not overweight (BMI < 25.0); overweight (25.0–29.9); obese (30.0 or more). For those with obesity, we also determined whether they lost weight to non-obesity or had weight loss of >5% [40] between the baseline and follow-up.

### Covariates

All covariates were measured at the baseline and were typically either self-reported or register-based depending on the study in question. Socioeconomic status was based on occupational position, or educational qualification and was categorised as high, intermediate, and low. Health-related factors included pre-existing chronic somatic disease (e.g., cardiovascular disease, diabetes, asthma, rheumatoid arthritis), and psychological distress or depressive symptoms. Smoking (current vs non-smoker) [41] and leisure-time physical activity (low vs intermediate/high) [42] were both self-reported.

### Statistical analyses

We used a two-stage approach to the present meta-analysis [43]. First, we estimated study-specific associations with individual participant data. With the outcomes of interest being relatively common, we used log-binomial regression analysis with relative risk (RR) as an estimator of effect and its 95% confidence intervals as an indicator of precision.

In the second stage, we used random effects meta-analysis to obtain a pooled estimate from the first-stage study-specific estimates. We conducted our analyses in seven parts. Part 1: forest plots including cohort-specific estimates from main analysis comparing different working hours categories in relation to weight gain—that is, transition from non-overweight to overweight/obesity), adjusted for age, sex and socioeconomic status. Significance of trend was assessed by meta-regression.

Further parts were conducted comparing ≥55 weekly working hours to 35–40 h. Part 2: weight gain outcome as in analysis 1 plus adjustment for chronic somatic disease, psychological distress/depressive symptoms, smoking and physical activity. Part 3: sensitivity analysis excluding smokers (adjusted for age, sex and socioeconomic status). Part 4: sensitivity analysis excluding those with chronic disease at baseline (adjusted for age, sex and socioeconomic status). Part 5: sensitivity analysis excluding those with underweight (BMI < 18.5, *n* = 1343; adjusted for age, sex and socioeconomic status). Part 6: subgroup analyses by sex, age groups (<50 years and ≥50 years), socioeconomic status group, and by follow-up time (1-2, 3-4 and 5–9 years).

Part 7: further transitions between BMI categories from baseline to follow-up: from non-overweight to overweight only; from overweight to obesity; and weight loss from obesity to non-obesity; and from obesity to >5% weight loss.

Heterogeneity of the study-specific estimates was assessed using the *I*^*2*^ statistic and τ [44] with a higher numerical value denoting greater heterogeneity. We calculated population attributable fraction (PAF) [18, 45] for overtime (>40) weekly hours, which indicates how much (in percentage) overweight/obesity in the general population would be reduced if all full-time workers worked standard 35–40 h, assuming that the associations are causal. The study-specific results were computed using SAS 9.4 (Cary, NC) and the meta-analyses were conducted using Stata 15 (College Station, TX).

## Results

Of the 122,078 participants, 61,143 (50.1%) were without overweight or obesity at baseline; 42,965 (35.2%) had overweight and 17,970 (14.7%) had obesity. Of those without overweight/obesity, 31,703 (51.9%) worked standard 35–40 h a week, 10,568 (17.3%) worked 41–48 h, 3897 (6.4%) worked 49–54 h and 3947 (6.5%) worked 55 h or more. The number of part-time workers was 11,028 (18.0%). Of the cohort, 12,349 (20.2%) gained weight and were overweight or obese at follow-up. The duration of follow-up across studies was 1–9 years (mean 4.4 years), corresponding to 267,491 person-years at risk for the participants without overweight/obesity (Supplementary Table 1).

**Fig. 1:**
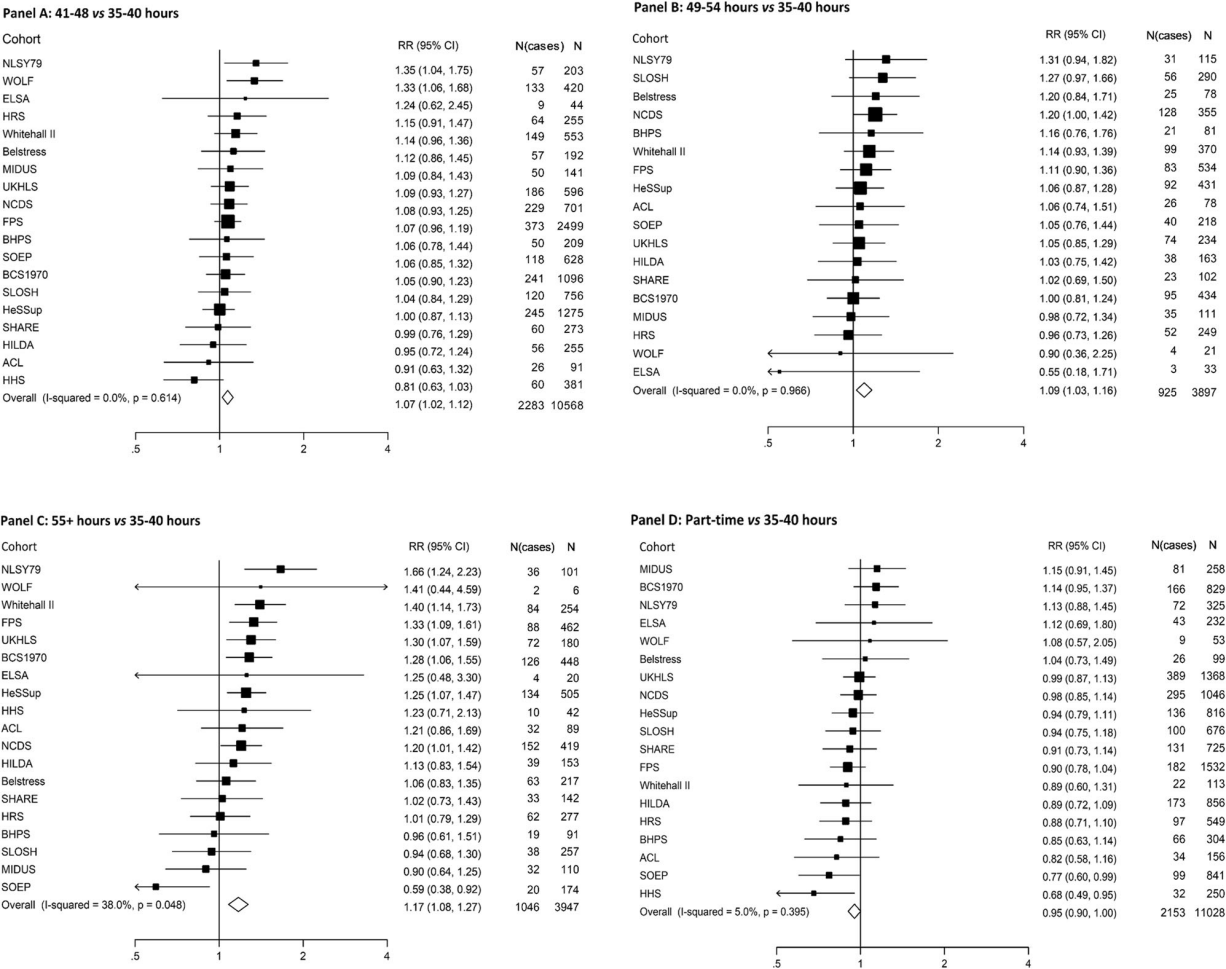
Relative risk (RR) and 95% confidence interval for the association between working hour category at baseline and onset of overweight/obesity at follow-up in participants with BMI <25 kg/m^2^ at baseline (random effects meta-analysis adjusted for age, sex and socioeconomic status) (**a**–**d**)

**Fig. 2:**
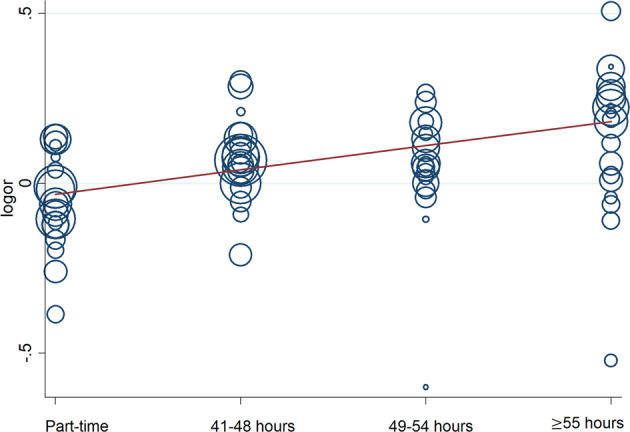
Illustration of a dose-response pattern (trend) on the association between working hours and onset of overweight/obesity from a meta-analysis of 19 studies

Figure 1a–d shows the forest plots of RRs for new-onset overweight/obesity at follow-up by the working hour categories. Compared with working standard 35–40 h a week, working 41–48 h was associated with RR = 1.07 (95% CI 1.02–1.12; Panel a); working 49–54 h with RR = 1.09 (95% CI 1.03–1.16; Panel b) and working 55 h or more with RR = 1.17 (95% CI 1.08–1.27; Panel c). Part-time work, in turn, was associated with RR = 0.95 (95% CI 0.90–1.00; Panel d). These findings suggest a dose-response relation between longer working hours and the risk of increasing body weight (*P* for trend <0.0001 in meta-regression; a graph presented in Fig. 2). Heterogeneity, as estimated by *I*^2^, suggested no (a, b, and d) or moderate (c) heterogeneity between study-specific estimates. *τ*-values in Panels a–d were 0, 0, 0.10 and 0.03, respectively.

**Fig. 3:**
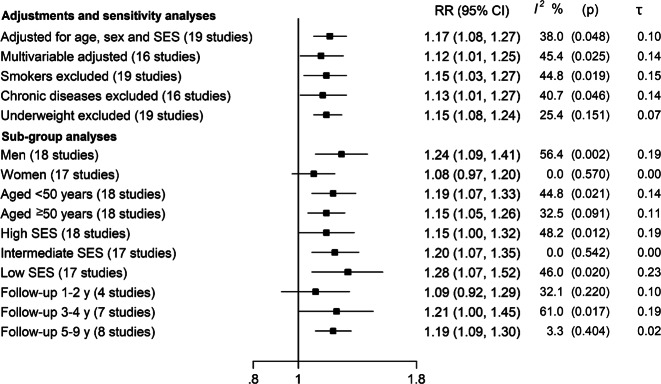
Summary relative risk (RR) of the onset of overweight/obesity for long (≥55 h/week) working hours at baseline compared with 35–40 h from serial adjustments and subgroup analyses in participants with BMI <25 kg/m^2^ at baseline. Relative risk ratios are adjusted for age, sex and socioeconomic status as appropriate

Multivariable adjustment for chronic somatic disease, mental health and lifestyle attenuated the association between long working hours and onset of overweight/obesity, from 1.17 (95% CI 1.08–1.27) to 1.12 (95% CI 1.01–1.25) (Fig. 3). Sensitivity analyses in which we excluded cigarette smokers, those with chronic somatic diseases, and those with underweight, had little impact on the associations. Similarly, results were unchanged following subgroup analyses in which we stratified the data by sex, age, socioeconomic group, or duration of follow-up. The greatest heterogeneity in effect estimates was evident in men, among those with high or low socioeconomic status, and in studies with medium-length follow-up (3–4 years). The overall PAF for overtime (>40 h) work was 2.8%, suggesting that if all full-time workers worked standard 35–40 h, the population level reduction of overweight/obesity would be 2.8%.

**Fig. 4:**
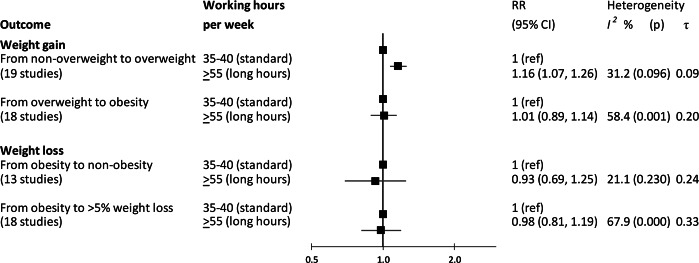
Summary relative risk of weight changes from baseline to follow-up for long (≥55 h/week) working hours at baseline compared with 35–40 working hours per week

Figure 4 shows the results of further analysis of the development of BMI between baseline and follow-up. Long (≥55) weekly working hours were associated with weight gain from non-overweight to overweight (RR = 1.16, 95% CI 1.07–1.26 and no significant heterogeneity), but not from overweight to obesity (RR = 1.01, 95% CI 0.89–1.14). No associations were observed between working hours and weight loss among participants with obesity.

## Discussion

In this individual-participant data meta-analysis of 19 cohort studies with 122,078 participants, we examined the prospective associations between working hours and weight change in a greater detail than has previously been possible. We found a modest association but a suggestive dose-response relation between longer working hours at baseline and the risk of weight gain from non-overweight to overweight or obesity at follow-up. The association with weekly working hours of 55 or more was further examined and the analyses suggest that the finding is robust to adjustments and replicable across subgroups. However, our detailed analysis of the development of BMI showed that long working hours may increase the risk of a shift from normal to overweight but not from overweight to the obese category. Working hours were not associated with weight loss among those who had obesity.

Our results are in line with previous prospective studies suggesting a positive association between long working hours and BMI [12–15] but in contrast with one small-scale Japanese study which reported that long working hours were associated with lower likelihood of weight gain [17]. The previous studies with positive findings were limited to datasets that included older [12] or younger [13] men and women, middle-aged women only [14], or industrial employees [15]. With a large dataset we were able to obtain more precise estimates for the association between working hours and BMI, including separate analyses for men and women and across age groups and socioeconomic strata.

The longest (≥55) weekly working hour category was associated with a 1.17-fold risk of new-onset overweight or obesity in the model adjusted for age, sex and socioeconomic status. The estimates from other working hour categories suggested a dose-response relationship although the overall association was modest. The association remained after adjustment for other lifestyle factors, chronic diseases and psychological distress, exclusion of smokers and those with chronic somatic disease or underweight, and in subgroup analyses by sex, age, socioeconomic status and length of follow-up. Thus, although weak, the association between long working hours and weight gain appeared to be robust. Our sensitivity analyses suggested that we can be most confident with the finding regarding a shift from normal to overweight category, with a RR of 1.16 with no significant heterogeneity in study-specific estimates.

Possible mechanism explaining the association between long working hours and weight gain may be extended periods of sitting, because today, many occupations are sedentary. Other mechanisms may be linked to too little exercise and unhealthy diet, because people with long working hours may not have resources, time or energy to engage in a healthy lifestyle [7]. Part-time workers—potentially having more time—seemed to have a lower risk of weight gain in our meta-analysis. This finding is in line with previous observations on Australian women who worked part time and had a smaller risk of weight gain than women who worked full time [14]. In our study, the 1.17-fold risk with long (≥55) weekly working hours attenuated to 1.12 in a multivariable adjusted model adding physical and mental health, smoking and physical inactivity. However, information on eating habits, one of the potential underlying mechanisms, was not available in our study. Another unmeasured mechanism could be prolonged sitting time at work, because our measures of physical activity covered only leisure-time physical inactivity. However, even though prolonged overall sitting time has been associated with a higher occupational status [46], our findings did not suggest differences the association between long working hours and weight gain between socioeconomic groups. Moreover, commuting hours would be an important factor to be considered in further studies to increase our understanding of the relationship between time-related factors and weight gain. However, previous studies have suggested that unhealthy lifestyle is not a major mechanism between long working hours and cardiovascular diseases [7]. The modest association found between long working hours and weight gain in the present study also supports the hypothesis of mechanisms being other than obesity.

Long working hours were not associated with weight loss among participants with obesity. However, we could not distinguish between intentional and unintentional weight loss. One could assume that psychosocial factors, such as work-related stress, affect success in intentional attempts to lose weight among overweight individuals whereas other factors, such as the onset of physical diseases may contribute to unintentional weight loss. However, the relationship between stress and weight change has been suggested to be complex as for some people stress seem to induce weight gain whereas for others it is associated with weight loss and for still others stress has no significant effect on weight [6].

Important strengths of our study include the first large-scale longitudinal individual participant data analysis of the association between working hours and weight change. A large sample size with harmonised variables reduces the likelihood of random error. We included cohort data from several countries in Europe, US and Australia, which increases generalisability of our findings, however, to those countries only. Notable limitations include an observational study design, which does not allow us to determine causality, and self-reported exposure to working hours and BMI in almost all cohorts. Working hours was measured only once although for some employees, long reported working hours may have had been a temporary ‘peak’ which was resolved shortly after the baseline survey whereas for others, excessive hours may have had been persistent. Self-report may also involve recall bias if the participants’ recall hours worked inaccurately or if they overestimate their height and underestimate their weight [47]. However, the validity of self-reported working hours has been found to be at least moderate [48] and the associations between BMI and cardiometabolic disease have been shown to be similar for self-reported and directly measured weight and height [49]. We were thus unable to assess whether the relatively small overall risk was an underestimate due to misclassification of the exposure or outcome. However, exposure misclassification may contribute to both under- and over-estimation of associations.

The PAF was 2.8% which suggests that overweight/obesity could be reduced by 2.8% if all full-time workers worked standard 35 to 40 h a week and the observed associations were causal. Given the multifactorial aetiology of obesity, this PAF is not trivial for population-wide prevention although the clinical significance at the individual level is modest. This size of PAF is comparable with those obtained for the relation of work stress (3.4%) [18] and depressive symptoms (1.1%) [45] with coronary heart disease although direct comparisons with BMI are not available. However, due to the causality assumption, findings on PAF in observational studies should be interpreted cautiously.

In conclusion, this individual participant meta-analysis of 19 cohort studies found a small excess risk of overweight among individuals who work longer hours. Detailed analyses were consistent with the hypothesis that the impact of long working hours involves the progression from normal to overweight rather than from overweight to obesity and that working long hours is not associated with weight loss among individuals with obesity. Future longitudinal studies assessing causality and underlying mechanisms, such as diet and accelerometer-measured physical activity would increase our understanding of the effects of long working hours on changes in body weight.

## Supplementary information

Supplementary Table 1
