# Supplementary material for: Long working hours and change in body weight: analysis of individual-participant data from 19 cohort studies
Source: Int J Obes (Lond). 2019 Nov 25;44(6):1368–75. doi: 10.1038/s41366-019-0480-3 (PMC7260128; doi:10.1038/s41366-019-0480-3)
Supplement: Supplementary file 1 — Supplementary Table 1 [file 41366_2019_480_MOESM1_ESM.docx]

**Supplementary Table 1**. Characteristics of participants without overweight/obesity at baseline in 19 cohort studies

| Cohort | Country | N of par-ticipants in main analysis | Mean follow-up (y) | Mean (SD) age (y) | N (%) women | N (%) with low SES | N (%) with chronic somatic disease | N (%) with psychological distress | N (%) smokers | N (%) with low physical activity | N (%) with overweight / obesity at follow-up |
| --- | --- | --- | --- | --- | --- | --- | --- | --- | --- | --- | --- |
| Americans’ Changing Lives (ACL)[^25^](#_ENREF_25) | USA | 715 | 3 | 41.9 (13.3) | 408 (57.1) | 249 (34.8) | 170 (23.9) | 101 (14.1) | 419 (58.6) | 131 (18.3) | 208 (29.1) |
| British Birth Cohort 1970 (BCS1970)[^19^](#_ENREF_19) | UK | 4065 | 4 | 29.8 (0.4) | 2277 (56.0) | 1167 (28.7) | – | 1157 (28.6) | 2162 (53.2) | 1112 (34.3) | 876 (21.5) |
| British Household Panel Survey (BHPS)[^20^](#_ENREF_20) | UK | 1040 | 2 | 42.3 (12.6) | 458 (44.0) | 191 (18.4) | 189 (18.2) | 209 (20.3) | 284 (27.3) | – | 240 (23.1) |
| German Socioeconomic Panel Survey (SOEP)[^35^](#_ENREF_35) | Germany | 2654 | 4 | 42.2 (11.5) | 1650 (62.2) | 491 (18.5) | 154 (5.8) | 401 (15.2) | 803 (30.3) | 577 (21.8) | 418 (15.7) |
| Household, Income and Labour Dynamics in Australia (HILDA)[^36^](#_ENREF_36) | Australia | 2050 | 4 | 39.9 (13.4) | 1184 (57.8) | 507 (24.7) | 220 (11.7) | 338 (17.2) | 391 (20.0) | 397 (20.2) | 449 (21.9) |
| Midlife in the United States (MIDUS)[^26^](#_ENREF_26) | USA | 1052 | 9 | 43.2 (11.0) | 695 (66.1) | 259 (24.6) | 351 (33.6) | 132 (12.5) | 249 (23.7) | 284 (27.0) | 329 (31.3) |
| National Child Development Study (NCDS)[^21^](#_ENREF_21) | UK | 3402 | 8 | 42.0 (0.0) | 1989 (58.5) | 1013 (29.8) | – | 637 (18.9) | 983 (28.9) | – | 1067 (31.4) |
| UK Household Longitudinal Study (UKHLS)[^23^](#_ENREF_23) | UK | 3383 | 1 | 42.2 (13.0) | 2105 (62.2) | 1096 (32.4) | – | 662 (21.5) | 711 (36.8) | – | 1011 (29.9) |
| English Longitudinal Study of Aging (ELSA)[^24^](#_ENREF_24) | UK | 490 | 4 | 58.1 (4.8) | 291 (59.4) | 140 (28.6) | 224 (45.7) | 48 (9.8) | 96 (19.6) | 41 (8.4) | 86 (17.6) |
| Health and Retirement Study (HRS)[^27^](#_ENREF_27) | USA | 2547 | 2 | 54.1 (5.4) | 1534 (60.2) | 732 (28.7) | 1078 (42.3) | 434 (17.0) | 797 (31.3) | 481 (18.9) | 538 (21.1) |
| Survey of Health, Ageing and Retirement in Europe (SHARE)[^37^](#_ENREF_37) | Europe | 1861 | 7 | 55.7 (5.7) | 1119 (60.1) | 306 (16.4) | 421 (22.6) | 331 (18.0) | 477 (25.6) | 278 (14.9) | 385 (20.7) |
| National Longitudinal Survey of Youth (NLSY79)[^28^](#_ENREF_28) | USA | 1600 | 4 | 41.0 (0.8) | 1008 (63.0) | 350 (21.9) | 355 (22.3) | 196 (12.3) | 484 (31.3) | 265 (16.6) | 374 (23.4) |
| Finnish Public Sector Study (FPS)[^29^](#_ENREF_29) | Finland | 18425 | 4 | 43.7 (9.2) | 16004 (86.9) | 2388 (13.0) | 4941 (27.1) | 4557 (24.8) | 2834 (15.8) | 2628 (14.3) | 2674 (14.5) |
| Helsinki Health Study (HHS)[^31^](#_ENREF_31) | Finland | 2779 | 7 | 48.7 (6.5) | 2340 (84.2) | 287 (10.3) | 688 (24.8) | 526 (19.0) | 592 (21.4) | 370 (13.3) | 532 (19.1) |
| Health and Social Support Study (HeSSup)[^30^](#_ENREF_30) | Finland | 7061 | 5 | 38.8 (10.1) | 4572 (64.8) | 824 (11.7) | 1669 (23.7) | 1084 (15.4) | 1659 (23.6) | 1169 (16.6) | 1373 (19.4) |
| Swedish Longitudinal Occupational Survey of Health (SLOSH)[^32^](#_ENREF_32) | Sweden | 3167 | 2 | 47.8 (10.8) | 2069 (65.3) | 770 (24.3) | 640 (20.5) | 141 (4.5) | 314 (9.9) | 404 (12.8) | 502 (15.9) |
| Whitehall II[^22^](#_ENREF_22) | UK | 2639 | 5 | 48.4 (5.8) | 800 (30.3) | 295 (11.2) | 224 (8.5) | 597 (22.6) | 317 (12.2) | 446 (16.9) | 672 (25.5) |
| Work, Lipids and Fibrinogen Norrland (WOLF)[^33^](#_ENREF_33) | Sweden | 918 | 5 | 41.6 (8.9) | 186 (20.3) | 557 (60.7) | 63 (7.0) | 5 (0.5) | 176 (19.7) | 203 (22.2) | 250 (27.2) |
| Belgian Job Stress Project (Belstress)[^34^](#_ENREF_34) | Belgium | 1295 | 7 | 42.4 (4.9) | 521 (40.2) | 414 (32.0) | 67 (5.2) | 264 (20.5) | 411 (32.2) | 220 (17.6) | 365 (28.2) |

Figures are n (%) except mean follow-up and mean age, years (SD=standard deviation). ‘–‘ = data not available.
